# Correction: The Sapir-Whorf Hypothesis and Probabilistic Inference: Evidence from the Domain of Color

**DOI:** 10.1371/journal.pone.0161521

**Published:** 2016-08-16

**Authors:** The PLOS ONE Staff

## Notice of Republication

This article was republished on August 5, 2016, to correct errors in Table 3 that occurred during composition of the article for publication. The colors displayed in the “Stimulus” column of Table 3 were incorrect. The publisher apologizes for the errors. Please download this article again to view the correct version. The originally published, uncorrected article and the republished, corrected article are provided here for reference.

## Supporting Information

S1 File Originally published, uncorrected article. (PDF)

S2 File Republished, corrected article. (PDF)
