# Supplementary material for: Correction: The Sapir-Whorf Hypothesis and Probabilistic Inference: Evidence from the Domain of Color
Source: PLoS One. 2016 Aug 16;11(8):e0161521. doi: 10.1371/journal.pone.0161521 (PMC4987024; doi:10.1371/journal.pone.0161521)
Supplement: S1 File — (PDF) [file pone.0161521.s001.PDF]

PLOS ONE

RESEARCH ARTICLE

The Sapir-Whorf Hypothesis and Probabilistic Inference: Evidence from the Domain of Color

Emily Cibelli[1☯], **Yang Xu**[2,3☯], **Joseph L. Austerweil**[4], **Thomas L. Griffiths**[3,5], **Terry Regier**[2,3]*

1 Department of Linguistics, Northwestern University, Evanston, IL 60208, United States of America, **2** Department of Linguistics, University of California, Berkeley, CA 94720, United States of America, **3** Cognitive Science Program, University of California, Berkeley, CA 94720, United States of America, **4** Department of Psychology, University of Wisconsin, Madison, WI 53706, United States of America, **5** Department of Psychology, University of California, Berkeley, CA 94720, United States of America

☯ These authors contributed equally to this work.
* terry.regier@berkeley.edu

CrossMark
click for updates

OPEN ACCESS

Citation: Cibelli E, Xu Y, Austerweil JL, Griffiths TL, Regier T (2016) The Sapir-Whorf Hypothesis and Probabilistic Inference: Evidence from the Domain of Color. PLoS ONE 11(7): e0158725. doi:10.1371/journal.pone.0158725

Editor: Daniel Osorio, University of Sussex, UNITED KINGDOM

Received: October 26, 2015

Accepted: June 21, 2016

Published: July 19, 2016

Copyright: © 2016 Cibelli et al. This is an open access article distributed under the terms of the Creative Commons Attribution License, which permits unrestricted use, distribution, and reproduction in any medium, provided the original author and source are credited.

Data Availability Statement: All relevant data are available within the paper and/or at: https://github.com/yangxuch/probwhorfcolor This GitHub repository is mentioned in the paper.

Funding: This research was supported by the National Science Foundation (www.nsf.gov) under grants DGE-1106400 (EC) and SBE-1041707 (YX, TR). Publication was made possible in part by support from the Berkeley Research Impact Initiative (BRII) sponsored by the UC Berkeley Library. The funders had no role in study design, data collection and analysis, decision to publish, or preparation of the manuscript.

Abstract

The Sapir-Whorf hypothesis holds that our thoughts are shaped by our native language, and that speakers of different languages therefore think differently. This hypothesis is controversial in part because it appears to deny the possibility of a universal groundwork for human cognition, and in part because some findings taken to support it have not reliably replicated. We argue that considering this hypothesis through the lens of probabilistic inference has the potential to resolve both issues, at least with respect to certain prominent findings in the domain of color cognition. We explore a probabilistic model that is grounded in a presumed universal perceptual color space and in language-specific categories over that space. The model predicts that categories will most clearly affect color memory when perceptual information is uncertain. In line with earlier studies, we show that this model accounts for language-consistent biases in color reconstruction from memory in English speakers, modulated by uncertainty. We also show, to our knowledge for the first time, that such a model accounts for influential existing data on cross-language differences in color discrimination from memory, both within and across categories. We suggest that these ideas may help to clarify the debate over the Sapir-Whorf hypothesis.

Introduction

The Sapir-Whorf hypothesis [1, 2] holds that our thoughts are shaped by our native language, and that speakers of different languages therefore think about the world in different ways. This proposal has been controversial for at least two reasons, both of which are well-exemplified in the semantic domain of color. The first source of controversy is that the hypothesis appears to undercut any possibility of a universal foundation for human cognition. This idea sits uneasily with the finding that variation in color naming across languages is constrained, such that

Competing Interests: The authors have declared that no competing interests exist.

certain patterns of color naming recur frequently across languages [3–5], suggesting some sort of underlying universal basis. The second source of controversy is that while some findings support the hypothesis, they do not always replicate reliably. Many studies have found that speakers of a given language remember and process color in a manner that reflects the color categories of their language [6–13]. Reinforcing the idea that language is implicated in these findings, it has been shown that the apparent effect of language on color cognition disappears when participants are given a verbal [7] (but not a visual) interference task [8, 11, 12]; this suggests that language may operate through on-line use of verbal representations that can be temporarily disabled. However, some of these findings have a mixed record of replication [14–17]. Thus, despite the substantial empirical evidence already available, the role of language in color cognition remains disputed.

An existing theoretical stance holds the potential to resolve both sources of controversy. On the one hand, it explains effects of language on cognition in a framework that retains a universal component, building on a proposal by Kay and Kempton [7]. On the other hand, it has the potential to explain when effects of language on color cognition will appear, and when they will not—and why. This existing stance is that of the "category adjustment" model of Huttenlocher and colleagues [18, 19]. We adopt this stance, and cast color memory as inference under uncertainty, instantiated in a category adjustment model, following Bae et al. [20] and Persaud and Hemmer [21]. The model holds that color memory involves the probabilistic combination of evidence from two sources: a fine-grained representation of the particular color seen, and the language-specific category in which it fell (e.g. English *green*). Both sources of evidence are represented in a universal perceptual color space, yet their combination yields language-specific bias patterns in memory, as illustrated in Fig 1. The model predicts that such category effects will be strongest when fine-grained perceptual information is uncertain. It thus has the potential to explain the mixed pattern of replications of Whorfian effects in the literature: non-replications could be the result of high perceptual certainty.

In the category adjustment model, both the fine-grained representation of the stimulus and the category in which it falls are modeled as probability distributions over a universal perceptual color space. The fine-grained representation is veridical (unbiased) but inexact: its distribution is centered at the location in color space where the stimulus itself fell, and the variance of that distribution captures the observer's uncertainty about the precise location of the stimulus in color space, with greater variance corresponding to greater uncertainty. Psychologically, such uncertainty might be caused by noise in perception itself, by memory decay over time, or by some other cause—and any increase in such uncertainty is modeled by a wider, flatter distribution for the fine-grained representation. The category distribution, in contrast, captures the information about stimulus location that is given by the named category in which the stimulus fell (e.g. *green* for an English-speaking observer). Because named color categories vary across languages, this category distribution is assumed to be language-specific—although the space over which it exists is universal. The model infers the original stimulus location by combining evidence from both of these distributions. As a result, the model tends to produce reconstructions of the stimulus that are biased away from the actual location of the stimulus and toward the prototype of the category in which it falls.

As illustrated in Fig 2, this pattern of bias pulls stimuli on opposite sides of a category boundary in opposite directions, producing enhanced distinctiveness for such stimuli. Such enhanced distinctiveness across a category boundary is the signature of categorical perception, or analogous category effects in memory. On this view, language-specific effects on memory can emerge from a largely universal substrate when one critical component of that substrate is language-specific: the category distribution.

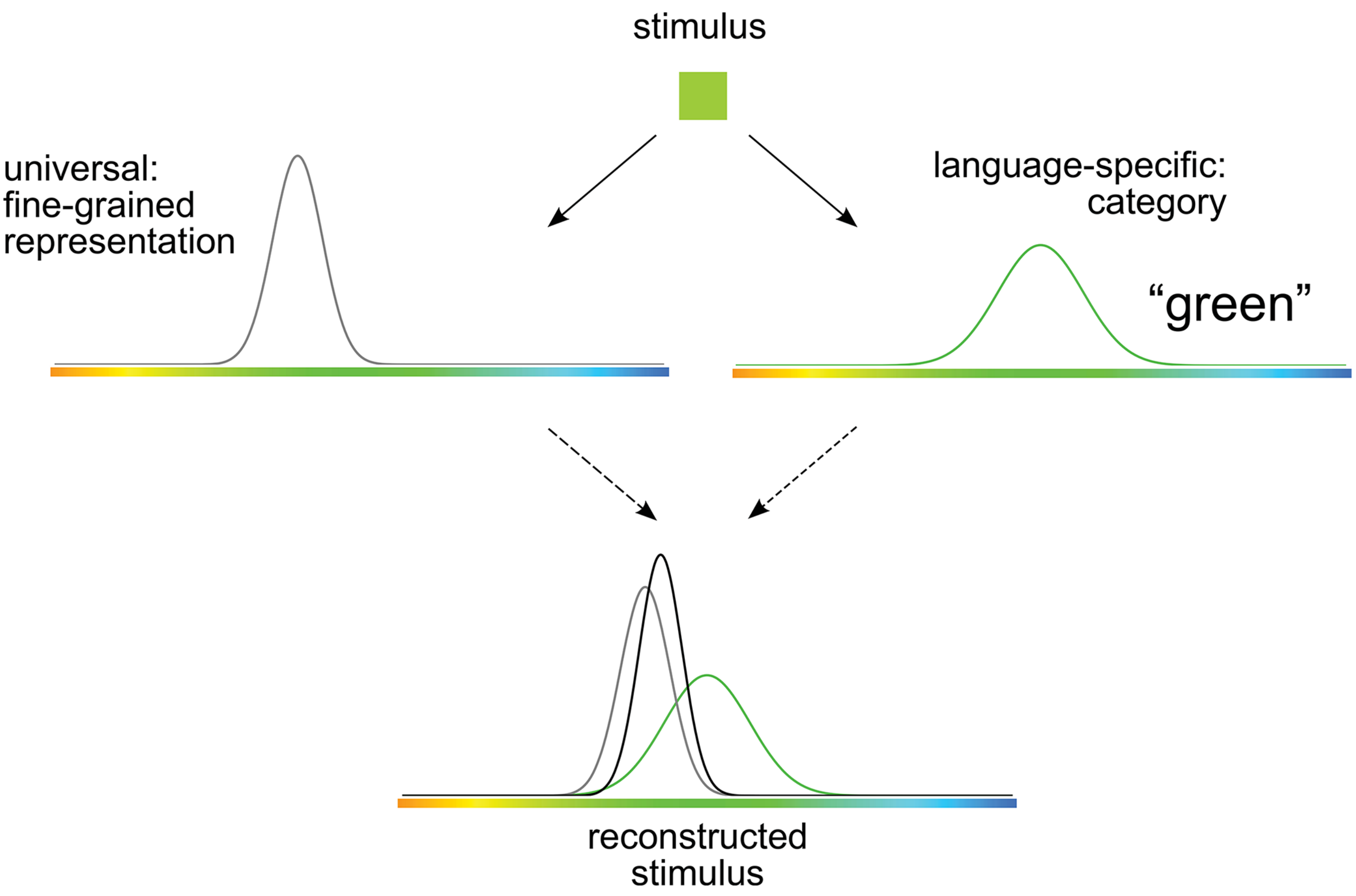

Fig 1. Model overview. A stimulus is encoded in two ways: (1) a fine-grained representation of the stimulus itself, shown as a (gray) distribution over stimulus space centered at the stimulus' location in that space, and (2) the language-specific category (e.g. English "green") in which the stimulus falls, shown as a separate (green) distribution over the same space, centered at the category prototype. The stimulus is reconstructed by combining these two sources of information through probabilistic inference, resulting in a reconstruction of the stimulus (black distribution) that is biased toward the category prototype. Adapted from Fig 11 of Bae et al. (2015) [20].

doi:10.1371/journal.pone.0158725.g001

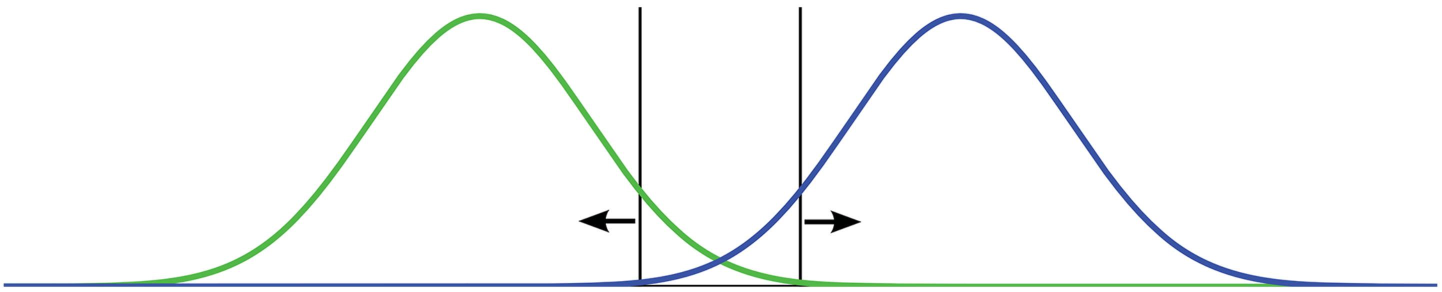

Fig 2. Category effects from biased reconstruction. Model reconstructions tend to be biased toward category prototypes, yielding enhanced distinctiveness for two stimuli that fall on different sides of a category boundary. Categories are shown as distributions in green and blue; stimuli are shown as vertical black lines; reconstruction bias patterns are shown as arrows.

doi:10.1371/journal.pone.0158725.g002

If supported, the category adjustment model holds the potential to clarify the debate over the Sapir-Whorf hypothesis in three ways. First, it would link that debate to independent principles of probabilistic inference. In so doing, it would underscore the potentially important role of *uncertainty*, whether originating in memory or perception, in framing the debate theoretically. Second, and relatedly, it would suggest a possible reason why effects of language on color memory and perception are sometimes found, and sometimes not [17]. Concretely, the model predicts that greater uncertainty in the fine-grained representation—induced for example through a memory delay, or noise in perception—will lead to greater influence of the category, and thus a stronger bias in reproduction. The mirror-image of this prediction is that in situations of relatively high certainty in memory or perception, there will be little influence of the category, to the point that such an influence may not be empirically detectable. Third, the model suggests a way to think about the Sapir-Whorf hypothesis without jettisoning the important idea of a universal foundation for cognition.

Closely related ideas appear in the literature on probabilistic cue integration [22–25]. For example, Ernst and Banks [24] investigated perceptual integration of cues from vision and touch in judging the height of an object. They found that humans integrate visual and haptic cues in a statistically optimal fashion, modulated by cue certainty. The category adjustment model we explore here can be seen as a form of probabilistic cue integration in which one of the cues is a language-specific category.

The category adjustment model has been used to account for category effects in various domains, including spatial location [18, 26], object size [19, 27], and vowel perception [28]. The category adjustment model also bears similarities to other theoretical accounts of the Sapir-Whorf hypothesis that emphasize the importance of verbal codes [7, 8], and the interplay of such codes with perceptual representations [29–31]. Prior research has linked such category effects to probabilistic inference, following the work of Huttenlocher and colleagues [18, 19]. Roberson and colleagues [32] invoked the category adjustment model as a possible explanation for categorical perception of facial expressions, but did not explore a formal computational model; Goldstone [33] similarly referenced the category adjustment model with respect to category effects in the color domain. Persaud and Hemmer [21, 34] explored bias in memory for color, and compared empirically obtained memory bias patterns from English speakers with results predicted by a formally specified category adjustment model, but did not link those results to the debate over the Sapir-Whorf hypothesis, and did not manipulate uncertainty. More recently, a subsequent paper by the same authors and colleagues [35] explored category-induced bias in speakers of another language, Tsimané, and did situate those results with respect to the Sapir-Whorf hypothesis, but again did not manipulate uncertainty. Most recently, Bae et al. [20] extensively documented bias in color memory in English speakers, modeled those results with a category-adjustment computational model, and did manipulate uncertainty—but did not explore these ideas relative to the Sapir-Whorf hypothesis, or to data from different languages.

In what follows, we first present data and computational simulations that support the recent finding that color memory in English speakers is well-predicted by a category adjustment model, with the strength of category effects modulated by uncertainty. We then show, to our knowledge for the first time, that a category adjustment model accounts for influential existing cross-language data on color that support the Sapir-Whorf hypothesis.

Analyses

In this section we provide general descriptions of our analyses and results. Full details are supplied in the section on Materials and Methods.

Study 1: Color reconstruction in English speakers

Our first study tests the core assumptions of the category adjustment model in English speakers. In doing so, it probes questions that were pursued by two studies that appeared recently, after this work had begun. Persaud and Hemmer [21] and Bae et al. [20] both showed that English speakers' memory for a color tends to be biased toward the category prototype of the corresponding English color term, in line with a category adjustment model. Bae et al. [20] also showed that the amount of such bias increases when subjects must retain the stimulus in memory during a delay period, compared to when there is no such delay, as predicted by the principles of the category adjustment model. In our first study, we consider new evidence from English speakers that tests these questions, prior to considering speakers of different languages in our following studies.

English-speaking participants viewed a set of hues that varied in small steps from dark yellow to purple, with most hues corresponding to some variety of either green or blue. We collected two kinds of data from these participants: bias data and naming data. Bias data were based on participants' non-linguistic reconstruction of particular colors seen. Specifically, for each hue seen, participants recreated that hue by selecting a color from a color wheel, either while the target was still visible (Fig 3A: simultaneous condition), or from memory after a short delay (Fig 3B: delayed condition). We refer to the resulting data as bias data, because we are interested in the extent to which participants' reconstructions of the stimulus color are biased away from the original target stimulus. Afterwards, the same participants indicated how good an example of English *green* (as in Fig 3C) and how good an example of English *blue* each hue was. We refer to these linguistic data as naming data.

Fig 4 shows both naming and bias data as a function of target hue. The top panel of the figure shows the naming data and also shows Gaussian functions corresponding to the English color terms *green* and *blue* that we fitted to the naming data. Bias data were collected for only a subset of the hues for which naming data were collected, and the shaded region in the top panel of Fig 4 shows that subset, relative to the full range of hues for naming data. We collected bias data only in this smaller range because we were interested specifically in bias induced by the two color terms *blue* and *green*, and colors outside the shaded region seemed to us to clearly show some influence of neighboring categories such as *yellow* and *purple*. The bottom panel of the figure shows the bias data, plotted relative to the prototypes (means) of the fitted Gaussian functions for *green* and *blue*. It can be seen that reconstruction bias appears to be stronger in the delayed than in the simultaneous condition, as predicted, and that—especially in the delayed condition—there is an inflection in the bias pattern between the two category prototypes, suggesting that bias may reflect the influence of each of the two categories. The smaller shaded region in this bottom panel denotes the subset of these hues that we subsequently analyzed statistically, and to which we fit models. We reduced the range of considered hues slightly further at this stage, to ensure that the range was well-centered with respect to the two relevant category prototypes, for *green* and *blue*, as determined by the naming data.

The absolute values (magnitudes) of the bias were analyzed using a 2 (condition: simultaneous vs. delayed) × 15 (hues) repeated measures analysis of variance. This analysis revealed significantly greater bias magnitude in the delayed than in the simultaneous condition. It also revealed that bias magnitude differed significantly as a function of hue, as well as a significant interaction between the factors of hue and condition. The blue stars in Fig 4 denote hues for which the difference in bias magnitude between the simultaneous and delayed conditions reached significance. The finding of greater bias magnitude in the delayed than in the simultaneous condition is consistent with the proposal that uncertainty is an important mediating factor in such category effects, as argued by Bae et al. [20]. It also suggests that some documented

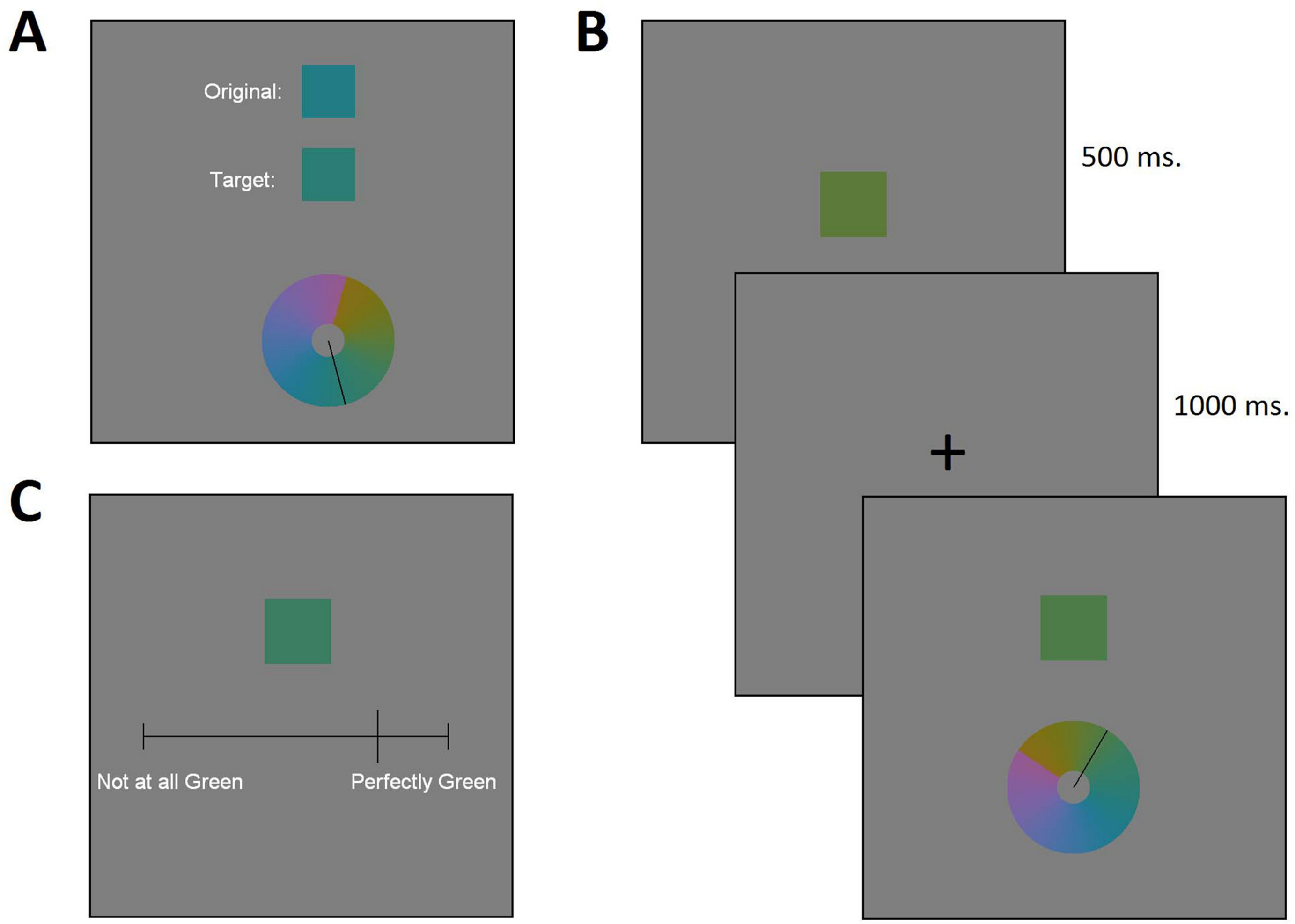

Fig 3. Screenshots of example trials illustrating (A) simultaneous reconstruction, (B) delayed reconstruction, and (C) *green* goodness rating.

doi:10.1371/journal.pone.0158725.g003

failures to find such category effects could in principle be attributable to high certainty, a possibility that can be explored by manipulating uncertainty.

We wished to test in a more targeted fashion to what extent these data are consistent with a category adjustment model in which a color is reconstructed based in part on English named color categories. To that end, we compared the performance of four models against these data; only one of these models considered both of the relevant English color categories, *green* and *blue*. As in Fig 1, each model contains a fine-grained but inexact representation of the perceived stimulus, and (for most models) a representation of one or more English color categories. Each model predicts the reconstruction of the target stimulus from its fine-grained representation of the target together with any category information. Category information in the model is specified by the naming data. Each model has a single free parameter, corresponding to the uncertainty of the fine-grained representation; this parameter is fit to bias data.

- The *null model* is a baseline model that predicts hue reconstruction based only on the fine-grained representation of the stimulus, with no category component.

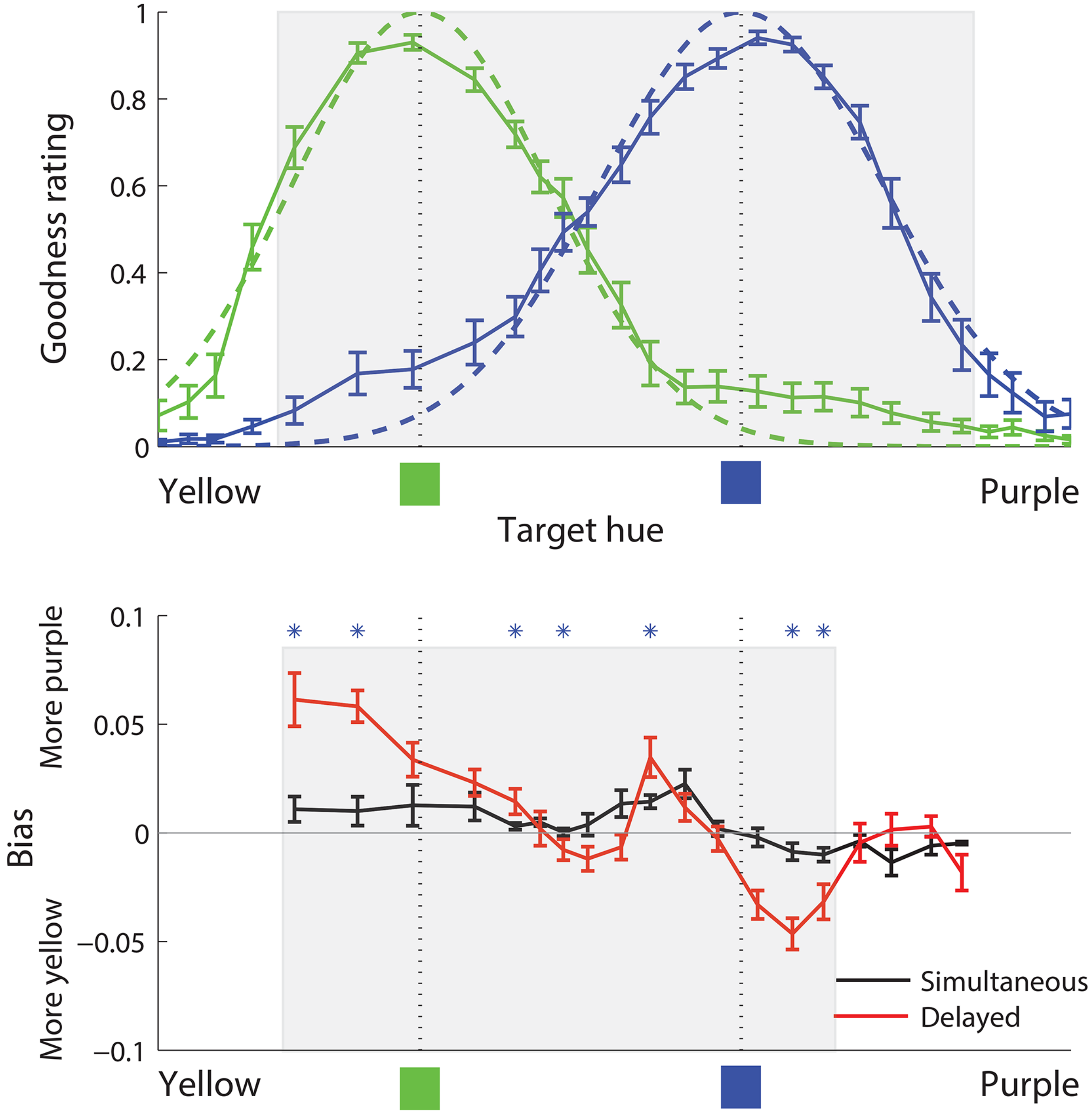

Fig 4. Naming and bias data, Study 1. In both top and bottom panels, the horizontal axis denotes target hue, ranging from yellow on the left to purple on the right. Top panel (naming data): The solid green and blue curves show, for each target hue, the average goodness rating for English *green* and *blue* respectively, as a proportion of the maximum rating possible. The dashed green and blue curves show Gaussian functions fitted to the naming goodness data. The dotted vertical lines marked at the bottom with green and blue squares denote the prototypes for *green* and *blue*, determined as the means of the green and blue fitted Gaussian functions, respectively. The shaded region in the top panel shows the portion of the spectrum for which bias data were collected. Bottom panel (bias data): Solid curves denote, for each target hue, the average reconstruction bias for that hue, such that positive values denote reconstruction bias toward the purple (here, right) end of the spectrum, and negative values denote reconstruction bias toward the yellow (here, left) end of the spectrum. Units for the vertical axis are the same as for the horizontal axis, which is normalized to length 1.0. The black and red curves show bias under simultaneous and delayed response, respectively. Blue stars at the top of the bottom panel mark hues for which there was a significant difference in the magnitude of bias between simultaneous and delayed conditions. The shaded region in the bottom panel shows the portion of the data that was analyzed statistically, and to which models were fit. In both panels, error bars represent standard error of the mean.

doi:10.1371/journal.pone.0158725.g004

- The *1-category (green) model* predicts hue reconstruction based on the fine-grained representation of the stimulus, combined with a representation of only the green category, derived from the *green* naming data.
- The *1-category (blue) model* predicts hue reconstruction based on the fine-grained representation of the stimulus, combined with a representation of only the blue category, derived from the *blue* naming data.
- The *2-category model* predicts hue reconstruction based on the fine-grained representation of the stimulus, combined with representations of both the *green* and *blue* categories.

If reproduction bias reflects probabilistic inference from a fine-grained representation of the stimulus itself, together with any relevant category, we would expect the 2-category model to outperform the others. The other models have access either to no category information at all (null model), or to category information for only one of the two relevant color categories (only one of *green* and *blue*). The 2-category model in contrast combines fine-grained stimulus information with both of the relevant categories (*green* and *blue*); this model thus corresponds most closely to a full category adjustment model.

Fig 5 redisplays the data from simultaneous and delayed reconstruction, this time with model fits overlaid. The panels in the left column show data from simultaneous reconstruction, fit by each of the four models, and the panels in the right column analogously show data and model fits from delayed reconstruction. Visually, it appears that in the case of delayed reconstruction, the 2-category model fits the data at least qualitatively better than competing models: it shows an inflection in bias as the empirical data do, although not as strongly. For simultaneous reconstruction, the 2-category model fit is also reasonable but visually not as clearly superior to the others (especially the null model) as in the delayed condition.

Table 1 reports quantitative results of these model fits. The best fit is provided by the 2-category model, in both the simultaneous and delayed conditions, whether assessed by log likelihood (LL) or by mean squared errror (MSE). In line with earlier studies [20, 21], these findings demonstrate that a category adjustment model that assumes stimulus reconstruction is governed by relevant English color terms provides a reasonable fit to data on color reconstruction by English speakers. The category adjustment model fits well both when the category bias is relatively slight (simultaneous condition), and when the bias is stronger (delayed condition).

Study 2: Color discrimination across languages

The study above examined the categories of just one language, English, whereas the Sapir-Whorf hypothesis concerns cross-language differences in categorization, and their effect on cognition and perception. Empirical work concerning this hypothesis has not specifically emphasized bias in reconstruction, but there is a substantial amount of cross-language data of other sorts against which the category adjustment model can be assessed. One method that has been extensively used to explore the Sapir-Whorf hypothesis in the domain of color is a two-alternative forced choice (2AFC) task. In such a task, participants first are briefly shown a target color, and then shortly afterward are shown that same target color together with a different distractor color, and are asked to indicate which was the color originally seen. A general finding from such studies [8–10] is that participants exhibit enhanced discrimination for pairs of colors that would be named differently in their native language. For example, in such a 2AFC task, speakers of English show enhanced discrimination for colors from the different English categories *green* and *blue*, compared with colors from the same category (either both *green* or both *blue*) [8]. In contrast, speakers of the Berinmo language, which has named color categories that differ from those of English, show enhanced discrimination across Berinmo category

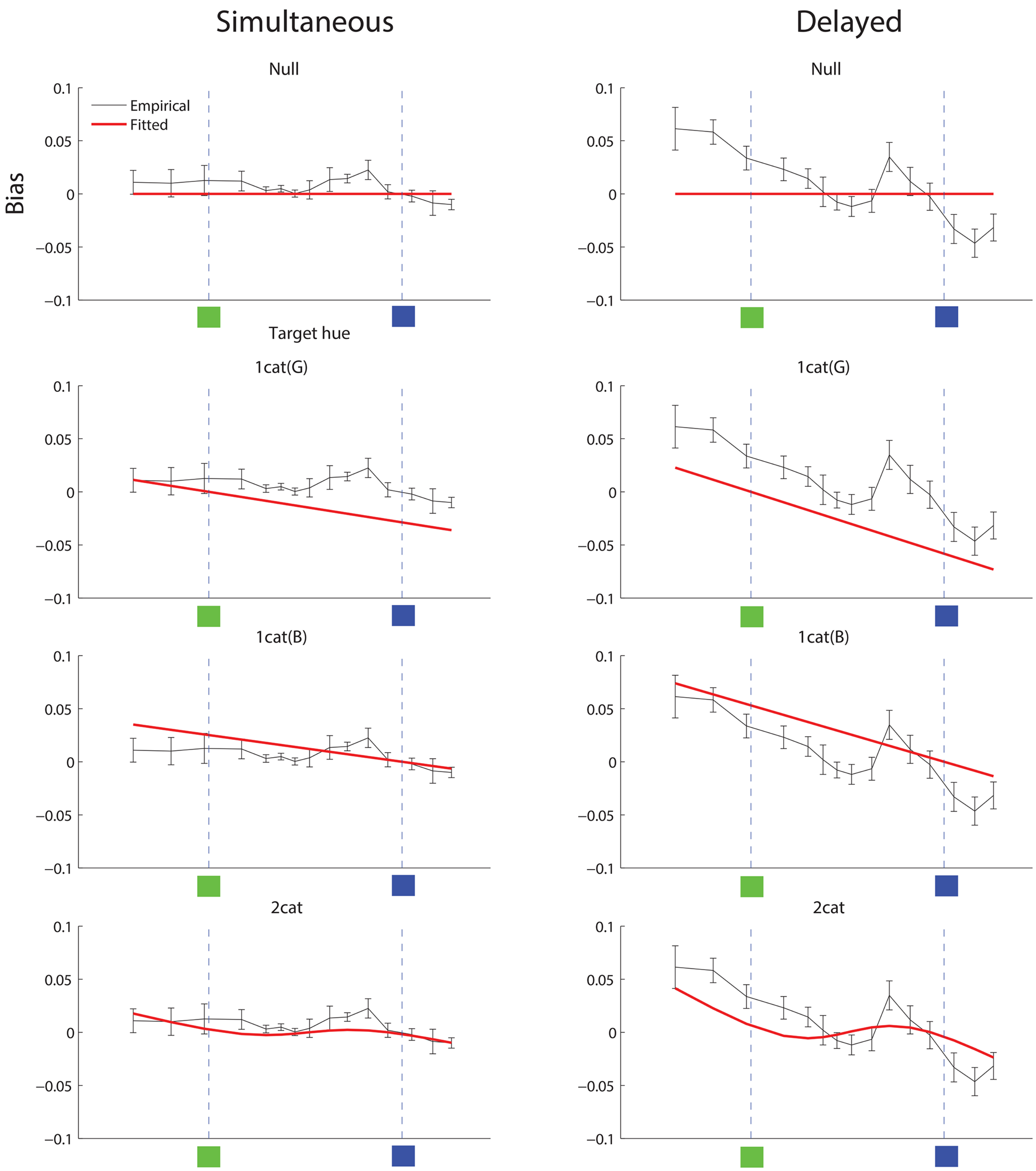
Simultaneous
Delayed
Null
1cat(G)
1cat(B)
2cat
Empirical
Fitted
Bias
Target hue
0.1
0.05
0
−0.05
−0.1

Fig 5. Bias in color reconstruction, and model fits, Study 1. Left column: Bias from simultaneous reconstruction, fit by each of the four models. The empirical data (black lines with error bars) in these four panels are the same, and only the model fits (red lines) differ. Within each panel, the horizontal axis denotes target hue, and the vertical axis denotes reconstruction bias. The green and blue prototypes are indicated as vertical lines with green and blue squares at the bottom. Right column: delayed reconstruction, displayed analogously.

doi:10.1371/journal.pone.0158725.g005

Table 1. Model fits to reconstruction data, Study 1. LL = log likelihood (higher is better). MSE = mean squared error (lower is better). The best value in each row is shown in **bold**.

	Measure	Null	1-cat. (G)	1-cat. (B)	2-cat.
Simultaneous	LL	2530	2290	2510	**2590**
	MSE	0.00011	0.00057	0.00015	**0.00008**
Delayed	LL	1860	1650	1900	**1980**
	MSE	0.00099	0.00160	0.00061	**0.00042**

doi:10.1371/journal.pone.0158725.t001

boundaries, and not across those of English [9]. Thus color discrimination in this task is enhanced at the boundaries of native language categories, suggesting an effect of those native language categories on the ability to discriminate colors from memory.

Considered informally, this qualitative pattern of results appears to be consistent with the category adjustment model, as suggested above in Fig 2. We wished to determine whether such a model would also provide a good *quantitative* account of such results, when assessed using the specific color stimuli and native-language naming patterns considered in the empirical studies just referenced.

We considered cross-language results from two previous studies by Debi Roberson and colleagues, one that compared color memory in speakers of English and Berinmo, a language of Papua New Guinea [9], and another that explored color memory in speakers of Himba, a language of Namibia [10]. Berinmo and Himba each have five basic color terms, in contrast with eleven in English. The Berinmo and Himba color category systems are similar to each other in broad outline, but nonetheless differ noticeably. Following these two previous studies, we considered the following pairs of categories in these three languages:

1. the English categories *green* and *blue*,
2. the Berinmo categories *wor* (covering roughly yellow, orange, and brown), and *nol* (covering roughly green, blue, and purple), and
3. the Himba categories *dumbu* (covering roughly yellow and beige) and *burou* (covering roughly green, blue, and purple).

These three pairs of categories are illustrated in Fig 6, using naming data from Roberson et al. (2000) [9] and Roberson et al. (2005) [10]. It can be seen that the English *green-blue* distinction is quite different from the Berinmo *wor-nol* and the Himba *dumbu-burou* distinctions, which are similar but not identical to each other. The shaded regions in this figure indicate specific colors that were probed in discrimination tasks. The shaded (probed) region that straddles a category boundary in Berinmo and Himba falls entirely within the English category *green*, and the shaded (probed) region that straddles a category boundary in English falls entirely within the Berinmo category *nol* and the Himba category *burou*, according to naming data in Fig 1 of Roberson et al. (2005) [10]. The empirical discrimination data in Fig 7 are based on those

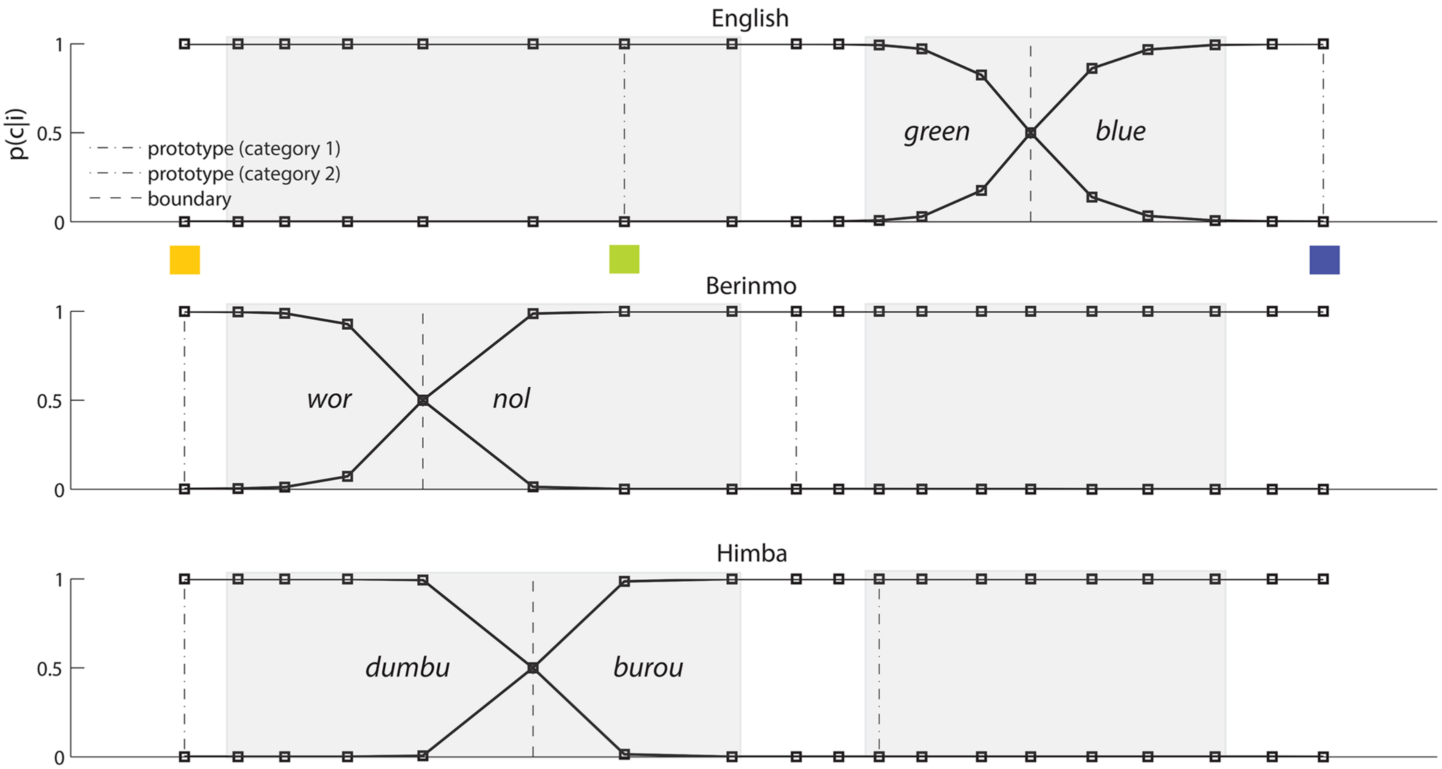

Fig 6. Color naming across languages, Study 2. The English categories *green* and *blue* (top panel), the Berinmo categories *wor* and *nol* (middle panel), and the Himba categories *dumbu* and *burou* (bottom panel), plotted against a spectrum of hues that ranges from dark yellow at the left, through green, to blue at the right. Colored squares mark prototypes: the shared prototype for Berinmo *wor* and Himba *dumbu*, and the prototypes for English *green* and *blue*; the color of each square approximates the color of the corresponding prototype. For each language, the dotted-and-dashed vertical lines denote the prototypes for the two categories from that language, and the dashed vertical line denotes the empirical boundary between these two categories. Black curves show the probability of assigning a given hue to each of the two native-language categories, according to the category component of a 2-category model fit to each language's naming data. The shaded regions mark the ranges of colors probed in discrimination tasks; these two regions are centered at the English *green-blue* boundary and the Berinmo *wor-nol* boundary. Data are from Roberson et al. (2000) [9] and Roberson et al. (2005) [10].

doi:10.1371/journal.pone.0158725.g006

probed colors [9, 10], and show that in general, speakers of a language tend to exhibit greater discrimination for pairs of colors that cross a category boundary in their native language, consistent with the Sapir-Whorf hypothesis.

We sought to determine whether the 2-category model explored above could account for these data. To that end, for each language, we created a version of the 2-category model based on the naming data for that language. Thus, we created an English model in which the two categories were based on empirical naming data for *green* and *blue*, a Berinmo model in which the two categories were based on empirical naming data for *wor* and *nol*, and a Himba model in which the two categories were based on empirical naming data for *dumbu* and *burou*. The black curves in Fig 6 show the probability of assigning a given hue to each of the two native-language categories, according to the category component of a 2-category model fit to each language's naming data. Given this category information, we simulated color reconstruction from memory for the specific colors considered in the empirical studies [9, 10] (the colors in the shaded regions in Fig 6). We did so separately for the cases of English, Berinmo, and Himba, in each case fitting a model based on naming data for a given language to discrimination data from speakers of that language. As in Study 1, we fit the model parameter corresponding to the

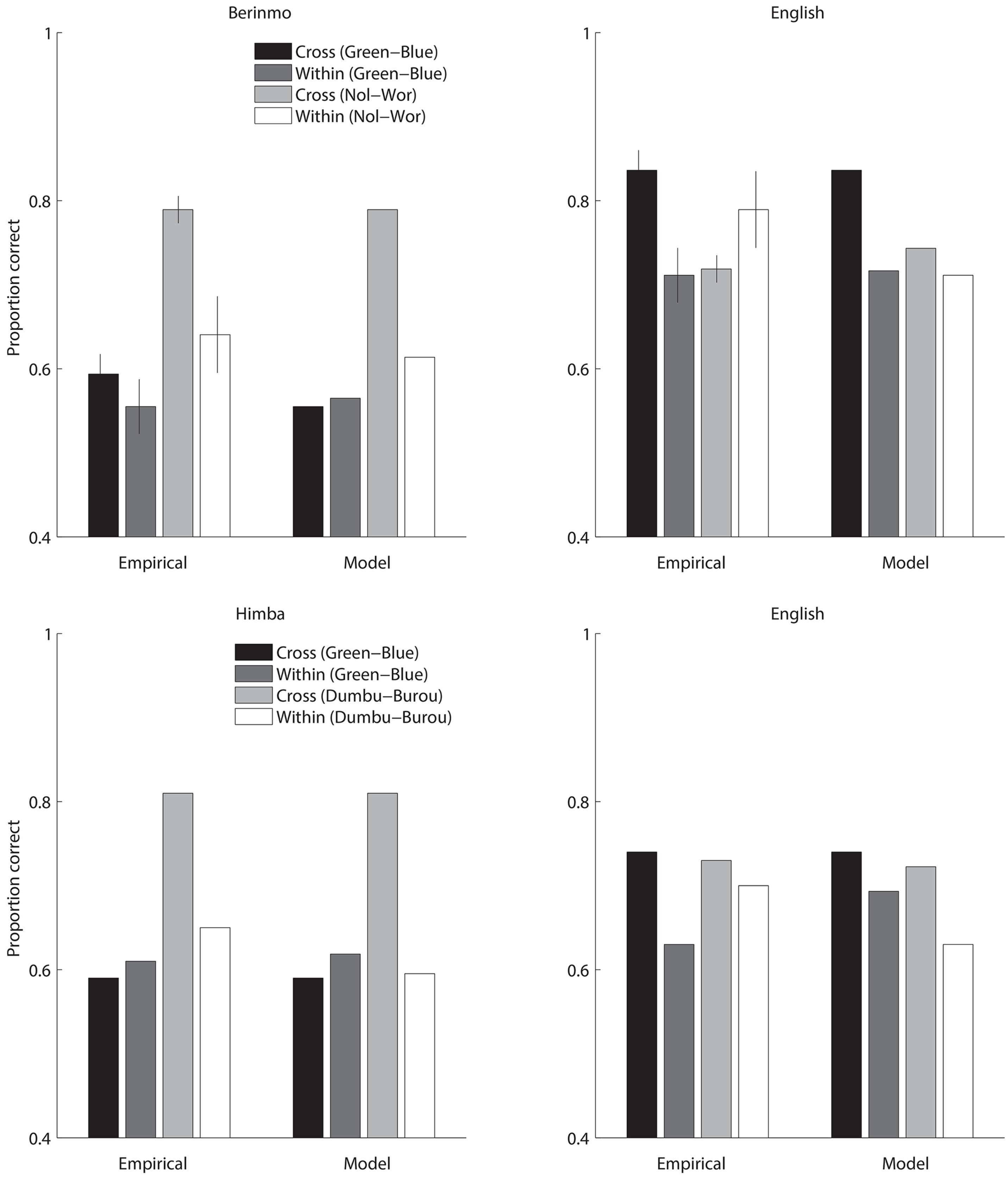
Berinmo
English
Himba
English
Proportion correct
Cross (Green–Blue)
Within (Green–Blue)
Cross (Nol–Wor)
Within (Nol–Wor)
Cross (Dumbu–Burou)
Within (Dumbu–Burou)
Empirical
Model
0.4
0.6
0.8
1

Fig 7. Color discrimination across languages, Study 2. Top panels: Discrimination from memory by Berinmo and English speakers for pairs of colors across and within English and Berinmo color category boundaries. Empirical data are from Table 11 of Roberson et al. (2000:392). Empirical values show mean proportion correct 2AFC memory judgments, and error bars show standard error. Model values show mean model proportion correct 2AFC memory judgments after simulated reconstruction with native-language categories. Model results are range-matched to the corresponding empirical values, such that the minimum and maximum model values match the minimum and maximum mean values in the corresponding empirical dataset, and other model values are linearly interpolated. Bottom panels: Discrimination from memory by Himba and English speakers for pairs of colors across and within English and Himba color category boundaries, compared with model results based on native-language categories. Empirical data are from Table 6 of Roberson et al. (2005:400); no error bars are shown because standard error was not reported in that table.

doi:10.1371/journal.pone.0158725.g007

uncertainty of fine-grained perceptual representation to the empirical non-linguistic (here discrimination) data, and we used a single value for this parameter across all three language models. The model results are shown in Fig 7, beside the empirical data to which they were fit. The models provide a reasonable match to the observed cross-language differences in discrimination. Specifically, the stimulus pairs for which empirical performance is best are those that cross a native-language boundary—and these are stimulus pairs for which the corresponding model response is strongest.

Although not shown in the figure, we also conducted a followup analysis to test whether the quality of these fits was attributable merely to model flexibility, or to a genuine fit between a language's category system and patterns of discrimination from speakers of that language. We did this by switching which language's model was fit to which language's discrimination data. Specifically, we fit the model based on Berinmo naming to the discrimination data from English speakers (and vice versa), and fit the model based on Himba naming to the discrimination data from English speakers (and vice versa), again adjusting the model parameter corresponding to the uncertainty of the fine-grained perceptual representation to the empirical discrimination data. The results are summarized in Table 2. It can be seen that the discrimination data are fit better by native-language models (that is, models with a category component originally fit to that language's naming data) than by other-language models (that is, models with a category component originally fit to another language's naming data). These results suggest that cross-language differences in discrimination may result from category-induced reconstruction bias under uncertainty, guided by native-language categories.

Study 3: Within-category effects

Although many studies of categorical perception focus on pairs of stimuli that cross category boundaries, there is also evidence for category effects *within* categories. In a 2AFC study of categorical perception of facial expressions, Roberson and colleagues [32] found the behavioral signature of categorical perception (or more precisely in this case, categorical memory):

Table 2. Model fits to cross-language discrimination data, Study 2, reported in mean squared error (lower is better). The best value in each row is shown in **bold**. Data are fit better by native-language models than by other-language models.

Berinmo-English comparison		
	Berinmo model	English model
Berinmo discrimination data	**0.0017**	0.0080
English discrimination data	0.0019	**0.0006**
Himba-English comparison		
	Himba model	English model
Himba discrimination data	**0.0022**	0.0039
English discrimination data	0.0029	**0.0008**

doi:10.1371/journal.pone.0158725.t002

superior discrimination for cross-category than for within-category pairs of stimuli. But in addition, they found an interesting category effect on within-category pairs, dependent on order of presentation. For each within-category pair they considered, one stimulus of the pair was always closer to the category prototype (the "good exemplar") than the other (the "poor exemplar"). They found that 2AFC performance on within-category pairs was better when the target was the good exemplar (and the distractor was therefore the poor exemplar) than when the target was the poor exemplar (and the distractor was therefore the good exemplar)—even though the same stimuli were involved in the two cases. Moreover, performance in the former (good exemplar) case did not differ significantly from cross-category performance. Hanley and Roberson [36] subsequently reanalyzed data from a number of earlier studies that had used 2AFC tasks to explore cross-language differences in color naming and cognition, including those reviewed and modeled in the previous section. Across studies and across domains, including color, they found the same asymmetrical within-category effect originally documented for facial expressions.

This within-category pattern may be naturally explained in category-adjustment terms, as shown in Fig 8, and as argued by Roberson and colleagues [32]. The central idea is that because the target is held in memory, it is subject to bias toward the prototype in memory, making discrimination of target from distractor either easier or harder depending on which of the two stimuli is the target. Although this connection with the category adjustment model has been made in the literature in general conceptual terms [32], followup studies have been theoretically focused elsewhere [31, 36], and the idea has not to our knowledge been tested computationally using the specific stimuli and naming patterns involved in the empirical studies. We sought to do so.

The empirical data in Fig 9 illustrate the within-category effect with published results on color discrimination by speakers of English, Berinmo, and Himba. In attempting to account for these data, we considered again the English, Berinmo, and Himba variants of the 2-category model first used in Study 2, and also retained from that study the parameter value corresponding to the uncertainty of the fine-grained perceptual representation, in the case of native-language models. We simulated reconstruction from memory of the specific colors examined in Study 2. Following the empirical analyses, this time we disaggregated the within-category stimulus pairs into those in which the target was a good exemplar of the category (i.e. the target was closer to the prototype than the distractor was), vs. those in which the target was a poor exemplar of the category (i.e. the target was farther from the prototype than the distractor was). The model results are shown in Fig 9, and match the empirical data reasonably well, supporting the informal in-principle argument of Fig 8 with a more detailed quantitative analysis.

Conclusions

We have argued that the debate over the Sapir-Whorf hypothesis may be clarified by viewing that hypothesis in terms of probabilistic inference. To that end, we have presented a probabilistic model of color memory, building on proposals in the literature. The model assumes both a universal color space and language-specific categorical partitionings of that space, and infers the originally perceived color from these two sources of evidence. The structure of this model maps naturally onto a prominent proposal in the literature that has to our knowledge not previously been formalized in these terms. In a classic early study of the effect of language on color cognition, Kay and Kempton [7] interpret Whorf [2] as follows:

> Whorf [. . .] suggests that he conceives of experience as having two tiers: one, a kind of rock bottom, inescapable seeing-things-as-they-are (or at least as human beings cannot help but

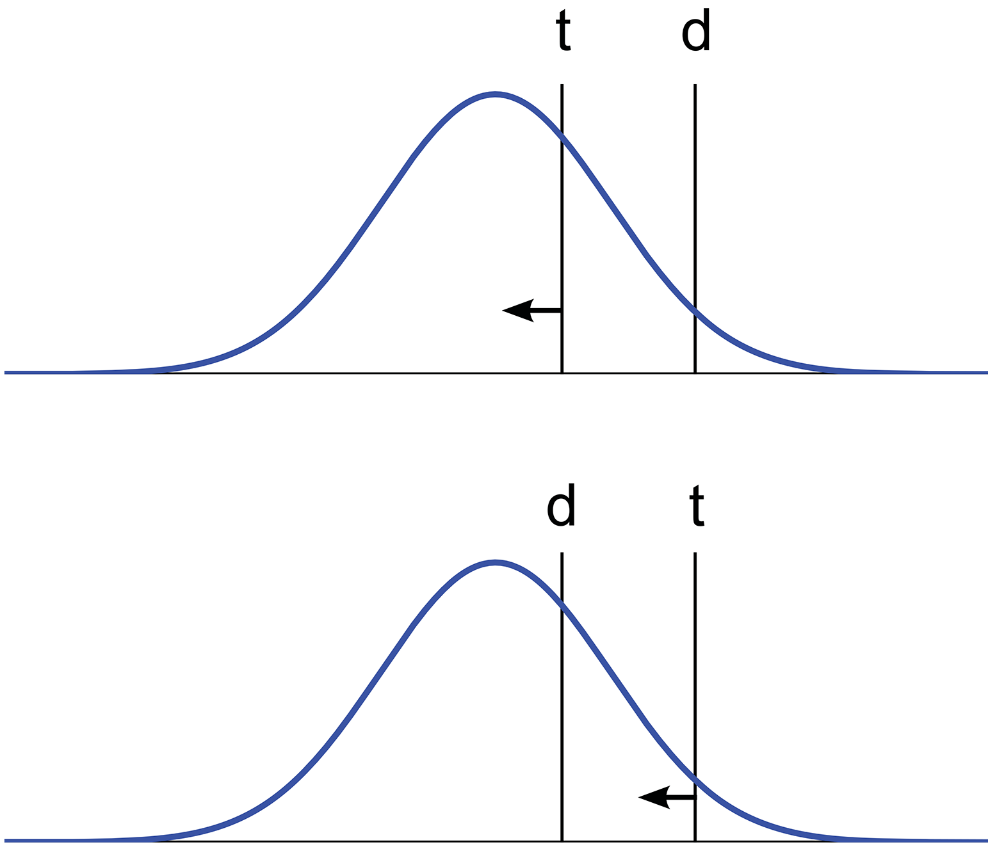

Fig 8. Within-category bias, dependent on presentation order. The category adjustment model predicts: (top panel, good exemplar) easy within-category discrimination in a 2AFC task when the initially-presented target t is closer to the prototype than the distractor d is; (bottom panel, poor exemplar) difficult within-category discrimination with the same two stimuli when the initially-presented target t is farther from the prototype than the distractor d is. Category is shown as a distribution in blue; stimuli are shown as vertical black lines marked t and d; reconstruction bias patterns are shown as arrows.

doi:10.1371/journal.pone.0158725.g008

> see them), and a second, in which [the specific structures of a given language] cause us to classify things in ways that could be otherwise (and are otherwise for speakers of a different language).

Kay and Kempton argue that color cognition involves an interaction between these two tiers. The existence of a universal groundwork for color cognition helps to explain why there are constraints on color naming systems across languages [3–5, 37]. At the same time, Kay and Kempton acknowledge a role for the language-specific tier in cognition, such that "there do appear to be incursions of linguistic categorization into apparently nonlinguistic processes of thinking" (p. 77). These two tiers map naturally onto the universal and language-specific components of the model we have explored here. This structure offers a straightforward way to think about effects of language on cognition while retaining the idea of a universal foundation underpinning human perception and cognition. Thus, this general approach, and our model as an instance of it, offer a possible resolution of one source of controversy surrounding the Sapir-Whorf hypothesis: taking that hypothesis seriously need not entail a wholesale rejection of important universal components of human cognition.

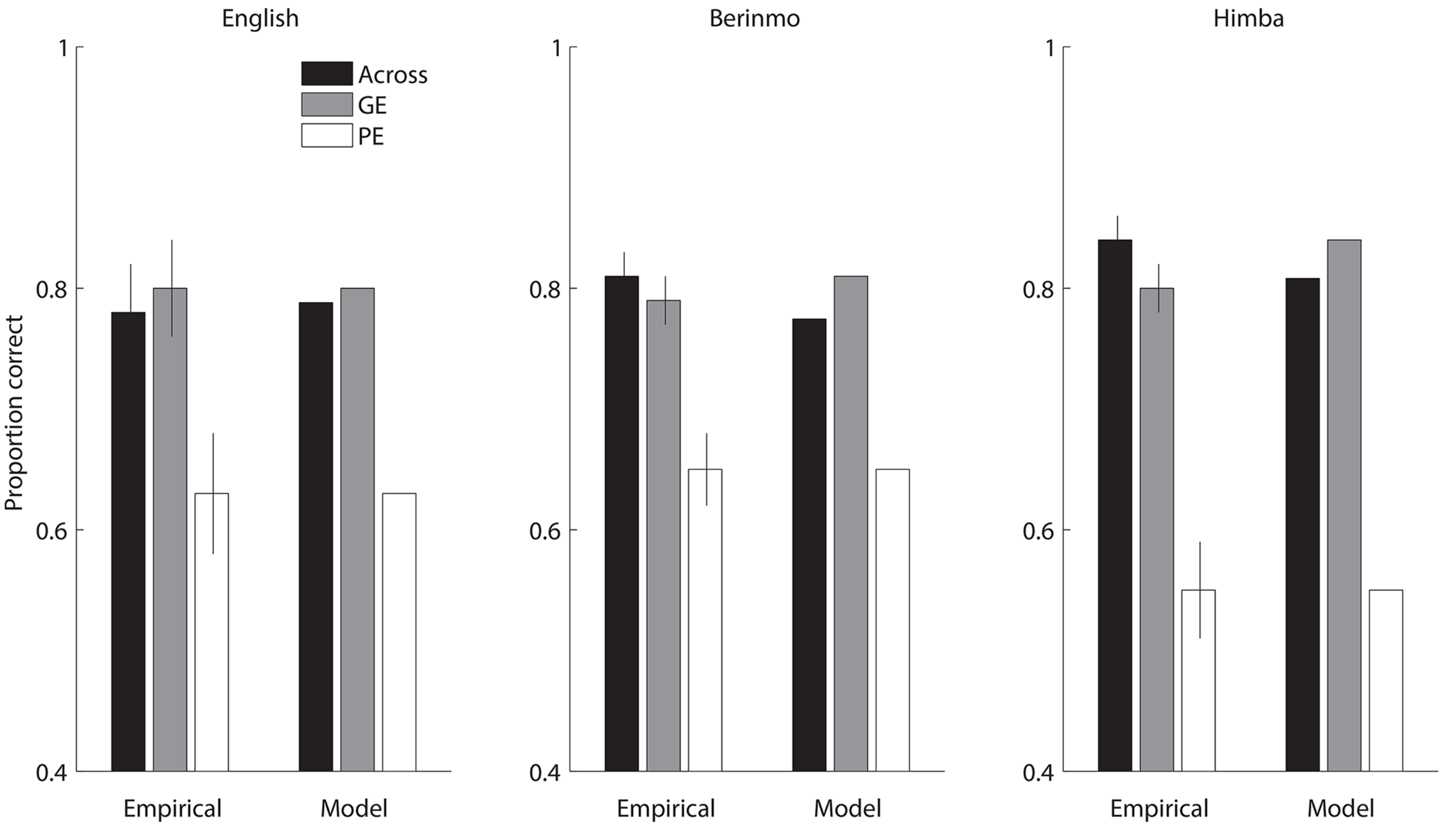

Fig 9. Within-category color discrimination across languages, Study 3. Across: stimulus pair crosses the native-language boundary; GE: within-category pair, target is the good exemplar; PE: within-category pair, target is the poor exemplar. Empirical data are from Figs 2 (English: 10-second retention interval), 3 (Berinmo), and 4 (Himba) of Hanley and Roberson [36]. Empirical values show mean proportion correct 2AFC memory judgments, and error bars show standard error. Model values show mean model proportion correct 2AFC memory judgments after simulated reconstruction using native-language categories, range-matched as in Fig 7. English model compared with English data: 0.00002 MSE; Berinmo model compared with Berinmo data: 0.00055 MSE; Himba model compared with Himba data: 0.00087 MSE.

doi:10.1371/journal.pone.0158725.g009

The approach proposed here also has the potential to resolve another source of controversy surrounding the Sapir-Whorf hypothesis: that some findings taken to support it do not replicate reliably (e.g. in the case of color: [15–17]). Framing the issue in terms of probabilistic inference touches this question by highlighting the theoretically central role of *uncertainty*, as in models of probabilistic cue integration [24]. We have seen stronger category-induced bias in color memory under conditions of greater delay and presumably therefore greater uncertainty (Study 1, and [20]). This suggests that in the inverse case of high certainty about the stimulus, any category effect could in principle be so small as to be empirically undetectable, a possibility that can be pursued by systematically manipulating uncertainty. Thus, the account advanced here casts the Sapir-Whorf hypothesis in formal terms that suggest targeted and quantitative followup tests. A related theoretical advantage of uncertainty is that it highlights an important level of generality: uncertainty could result from memory, as explored here, but it could also result from noise or ambiguity in perception itself, and on the view advanced here, the result should be the same.

The model we have proposed does not cover all aspects of language effects on color cognition. For example, there are documented priming effects [31] which do not appear to flow as

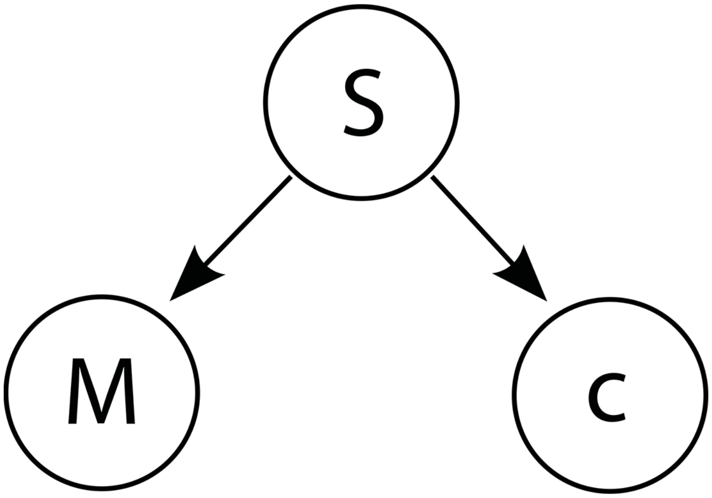

Fig 10. Model. The perception of stimulus $S = s$ produces a fine-grained memory M, and a categorical code c specifying the category in which s fell. We wish to reconstruct the original stimulus $S = s$, given M and c.

doi:10.1371/journal.pone.0158725.g010

naturally from this account as do the other effects we have explored above. However, the model does bring together disparate bodies of data in a simple framework, and links them to independent principles of probabilistic inference. Future research can usefully probe the generality and the limitations of the ideas we have explored here.

Materials and Methods

Code and data supporting the analyses reported here are available at https://github.com/yangxuch/probwhorfcolor.git.

Models

The basic model we consider is shown in Fig 10, which presents in graphical form the generative process behind Fig 1 above. Our model follows in general outline that of Bae et al. [20], but the formalization of inference within this structure more closely follows Feldman et al.'s [28] model of category effects in vowel perception. In our model, the perception of a stimulus $S = s$ produces a fine-grained memory M, and a categorical code c. We wish to obtain a reconstruction $\hat{s}$ of the original stimulus $S = s$, by combining evidence from the two internal representations M and c that s has produced. That reconstruction is derived as follows:

$$p(S|M,c) \propto p(M|S,c)p(S|c) \qquad \text{[Bayes' rule]} \tag{1}$$

$$\propto p(M|S)p(S|c) \qquad \text{[because } M \text{ is independent of } c \text{ given } S\text{]} \tag{2}$$

Because hue is a circular dimension, the components $p(M|S)$ and $p(S|c)$ could be modeled using circular normal or von Mises distributions, as was done by Bae et al. [20]. However each of our studies treats only a restricted subsection of the full hue circle, and for that reason we instead model these representations using normal distributions.

$p(M|S)$ represents the fine-grained memory trace M of the original stimulus $S = s$. We model this as a normal distribution with mean μ_m at the location of the original stimulus s, and

with uncertainty captured by variance σ_m^2:

$$p(M|S) = \mathcal{N}(M; \mu_m, \sigma_m^2) \tag{3}$$

This is an unbiased representation of the original stimulus s because $\mu_m = s$.

$p(S|c)$ captures the information about the location of stimulus S that is given by the categorical code c. We again model this as a normal distribution, this time centered at the prototype μ_c of category c, with variance σ_c^2:

$$p(S|c) = \mathcal{N}(S; \mu_c, \sigma_c^2) \tag{4}$$

This assumes that there is a single categorical code c, and we use this assumption in some of our model variants below. However in other cases we will capture the fact that more than one category may be applicable to a stimulus. In such cases we assume that the perceiver knows, for each category c, the applicability $\pi(c)$ of that category for the observed stimulus s. We model this as:

$$\pi(c) = p(c|s) \propto p(S = s|c)p(c) \tag{5}$$

where $p(S = s|c)$ is given by Eq (4) above, and $p(c)$ is assumed to be uniform.

We consider three variants of this basic model, described below in order of increasing complexity: the *null* model, the *1-category* model, and the *2-category* model. For each model, we take the predicted reconstruction $\hat{s}$ of a given stimulus $S = s$ to be the expected value of the posterior distribution:

$$\hat{s} = E[S|M, c] \tag{6}$$

Null model. The null model assumes that reconstruction is based only on the fine-grained memory, with no category influence. This model is derived from Eq (2) by assuming that the memory component $p(M|S)$ is as defined above, and the category component $p(S|c)$ is uniform, yielding:

$$p(S|M, -) \propto \mathcal{N}(M; \mu_m, \sigma_m^2) \tag{7}$$

The predicted reconstruction for this model is given by the expected value of this distribution, namely:

$$E[S|M, -] = \mu_m \tag{8}$$

where we have assumed $\mu_m = s$, the originally observed stimulus. This model predicts no category-induced bias: the reconstruction of the stimulus $S = s$ is simply the value of the stimulus s itself.

1-category model. The 1-category model assumes that reconstruction is based both on fine-grained memory and on information from a single category, e.g. English *green*. This model is derived from Eq (2) by assuming that both the memory component $p(M|S)$ and the category component $p(S|c)$ are as defined above, yielding:

$$p(S|M, c) \propto \mathcal{N}(M; \mu_m, \sigma_m^2)\, \mathcal{N}(S; \mu_c, \sigma_c^2) \tag{9}$$

The predicted reconstruction for this model is given by the expected value of this distribution, namely:

$$E[S|M, c] = \frac{\sigma_c^2}{\sigma_c^2 + \sigma_m^2}\mu_m + \frac{\sigma_m^2}{\sigma_c^2 + \sigma_m^2}\mu_c \tag{10}$$

where we have assumed $\mu_m = s$, the originally observed stimulus. This equation parallels Eq (7) of Feldman et al. [28]. This model produces a reconstruction that is a weighted average of the original stimulus value s and the category prototype μ_c, with weights determined by the relative certainty of each of the two sources of information. The same weighted average is also central to Ernst and Banks' [24] study of cue integration from visual and haptic modalities. That study was based on the same principles we invoke here, and our model—like that of Feldman et al.—can be viewed as a probabilistic cue integration model in which one of the two cues being integrated is a category, rather than a cue from a different modality.

2-category model. The 2-category model is similar to the 1-category model, but instead of basing its reconstruction on a single category c, it bases its reconstruction on two categories c_1 and c_2 (e.g. English *green* and *blue*). It does so by averaging together the reconstruction provided by the 1-category model for c_1 and the reconstruction provided by the 1-category model for c_2, weighted by the applicability $\pi(c)$ of each category c to the stimulus:

$$p(S|M) = \sum_{c \in \{c_1, c_2\}} p(S|M, c)\pi(c) \tag{11}$$

Here, $p(S|M, c)$ inside the sum is given by the 1-category model specified in Eq (9), and $\pi(c)$ is the applicability of category c to the observed stimulus s as specified above in Eq (5). Our equation here parallels Eq (9) of Feldman et al. [28] who similarly take a weighted average over 1-category models in their model of category effects in speech perception. The predicted reconstruction for this model is given by the expected value of this distribution, namely:

$$E[S|M] = \sum_{c \in \{c_1, c_2\}} \left(\frac{\sigma_c^2}{\sigma_c^2 + \sigma_m^2} \mu_m + \frac{\sigma_m^2}{\sigma_c^2 + \sigma_m^2} \mu_c \right) \pi(c) \tag{12}$$

assuming as before that $\mu_m = s$, the original stimulus value. This equation follows Feldman et al. [28] Eq (10).

Fitting models to data. For each model, any category parameters μ_c and σ_c^2 are first fit to naming data. The single remaining free parameter σ_m^2, corresponding to the uncertainty of fine-grained memory, is then fit to non-linguistic bias or discrimination data, with no further adjustment of the category parameters. Although this two-step process is used in all of our studies, it is conducted in slightly different ways across studies; we supply study-specific details below in our presentation of each study. All model fits were done using `fminsearch` in Matlab.

Study 1: Color reconstruction in English speakers

Participants. Twenty subjects participated in the experiment, having been recruited at UC Berkeley. All subjects were at least 18 years of age, native English speakers, and reported normal or corrected-to-normal vision, and no colorblindness. All subjects received payment or course credit for participation.

Informed consent was obtained verbally; all subjects read an approved consent form and verbally acknowledged their willingness to participate in the study. Verbal consent was chosen because the primary risk to subjects in this study was for their names to be associated with their response; this approach allowed us to obtain consent and collect data without the need to store subjects' names in any form. Once subjects acknowledged that they understood the procedures and agreed to participate by stating so to the experimenter, the experimenter recorded their consent by assigning them a subject number, which was anonymously linked to their data. All study procedures, including those involving consent, were overseen and approved by the UC Berkeley Committee for the Protection of Human Subjects.

Table 3. Stimulus coordinates, and approximate rendering of stimuli. All stimuli were presented at lightness 6, saturation 8 in Munsell space.

	Munsell hue	xyY coordinates	Stimulus
1	5Y	0.4426, 0.4588, 30.05	
2	7.5Y	0.4321, 0.4719, 30.05	
3	10Y	0.4201, 0.4812, 30.05	
4	2.5GY	0.4006, 0.4885, 30.05	
5	5GY	0.3772, 0.4880, 30.05	
6	7.5GY	0.3418, 0.4768, 30.05	
7	10GY	0.3116, 0.4563, 30.05	
8	2.5G	0.2799, 0.4239, 30.05	
9	5G	0.2612, 0.3990, 30.05	
10	7.5G	0.2510, 0.3829, 30.05	
11	10G	0.2420, 0.3679, 30.05	
12	2.5BG	0.2332, 0.3522, 30.05	
13	5BG	0.2236, 0.3311, 30.05	
14	7.5BG	0.2171, 0.3138, 30.05	
15	10BG	0.2116, 0.2950, 30.05	
16	2.5B	0.2080, 0.2789, 30.05	
17	5B	0.2088, 0.2635, 30.05	
18	7.5B	0.2132, 0.2537, 30.05	
19	10B	0.2189, 0.2468, 30.05	
20	2.5PB	0.2274, 0.2406, 30.05	
21	5PB	0.2360, 0.2365, 30.05	
22	7.5PB	0.2505, 0.2347, 30.05	
23	10PB	0.2637, 0.2352, 30.05	
24	2.5P	0.2770, 0.2372, 30.05	
25	5P	0.2905, 0.2421, 30.05	
26	7.5P	0.3099, 0.2502, 30.05	
27	10P	0.3259, 0.2584, 30.05	

doi:10.1371/journal.pone.0158725.t003

Stimuli. Stimuli were selected by varying a set of hues centered around the *blue-green* boundary, holding saturation and lightness constant. Stimuli were defined in Munsell coordinate space, which is widely used in the literature we engage here (e.g. [9, 10]). All stimuli were at lightness 6 and saturation 8. Hue varied from 5Y to 10P, in equal hue steps of 2.5. Colors were converted to xyY coordinate space following Table I(6.6.1) of Wyszecki and Stiles (1982) [38]. The colors were implemented in Matlab in xyY; the correspondence of these coordinate systems in the stimulus set, as well as approximate visualizations of the stimuli, are reported in Table 3.

We considered three progressively narrower ranges of these stimuli for different aspects of our analyses, in an attempt to focus the analyses on a region that is well-centered relative to the English color categories *green* and *blue*. We refer to these three progressively narrower ranges as the *full range*, the *medium range*, and the *focused range*. We specify these ranges below, together with the aspects of the analysis for which each was used.

- Full range: We collected naming data for *green* and *blue* relative to the full range, stimuli 1-27, for a total of 27 stimuli. We fit the category components of our models to naming data over this full range.

- Medium range: We collected bias data for a subset of the full range, namely the medium range, stimuli 5-23, for a total of 19 stimuli. We considered this subset because we were interested in bias induced by the English color terms *green* and *blue*, and we had the impression, prior to collecting naming or bias data, that colors outside this medium range had some substantial element of the neighboring categories *yellow* and *purple*.
- Focused range: Once we had naming data, we narrowed the range further based on those data, to the focused range, stimuli 5-19, for a total of 15 stimuli. The focused range extends between the (now empirically assessed) prototypes for *green* and *blue*, and also includes three of our stimulus hues on either side of these prototypes, yielding a range well-centered relative to those prototypes, as can be seen in the bottom panel of Fig 4 above. We considered this range in our statistical analyses, and in our modeling of bias patterns.

Experimental procedure. The experiment consisted of four blocks. The first two blocks were reconstruction (bias) tasks: one simultaneous block and one delay block. In the simultaneous block (Fig 3A), the subject was shown a stimulus color as a colored square (labeled as "Original" in the figure), and was asked to recreate that color in a second colored square (labeled as "Target" in the figure) as accurately as possible by selecting a hue from a color wheel. The ("Original") stimulus color remained on screen while the subject selected a response from the color wheel; navigation of the color wheel would change the color of the response ("Target") square. The stimulus square and response square each covered 4.5 degrees of visual angle, and the color wheel covered 11.1 degrees of visual angle. Target colors were drawn from the medium range of stimuli (stimuli 5—23 of Table 3). The color wheel was constructed based on the full range of stimuli (stimuli 1—27 of Table 3), supplemented by interpolating 25 points evenly in xyY coordinates between each neighboring pair of the 27 stimuli of the full range, to create a finely discretized continuum from yellow to purple, with 677 possible responses. Each of the 19 target colors of the medium range was presented five times per block in random order, for a total of 95 trials per block. The delay block (Fig 3B) was similar to the simultaneous block but with the difference that the stimulus color was shown for 500 milliseconds then disappeared, then a fixation cross was shown for 1000 milliseconds, after which the subject was asked to reconstruct the target color from memory, again using the color wheel to change the color of the response square. The one colored square shown in the final frame of Fig 3B is the response square that changed color under participant control. The order of the simultaneous block and delay block were counterbalanced by subject. Trials were presented with a 500 millisecond inter-trial interval.

Several steps were taken to ensure that responses made on the color wheel during the reconstruction blocks were not influenced by bias towards a particular spatial position. The position of the color wheel was randomly rotated up to 180 degrees from trial to trial. The starting position of the cursor was likewise randomly generated for each new trial. Finally, the extent of the spectrum was jittered one or two stimuli (2.5 or 5 hue steps) from trial to trial, which had the effect of shifting the spectrum slightly in the yellow or the purple direction from trial to trial. This was done to ensure that the *blue-green* boundary would not fall at a consistent distance from the spectrum endpoints on each trial.

The second two blocks were naming tasks. In each, subjects were shown each of the 27 stimuli of the full range five times in random order, for a total of 135 trials per block. On each trial, subjects were asked to rate how good an example of a given color name each stimulus was. In one block, the color name was *green*, in the other, the color name was *blue*; order of blocks was counterbalanced by subject. To respond, subjects positioned a slider bar with endpoints "Not at all [green/blue]" and "Perfectly [green/blue]" to the desired position matching their judgment of each stimulus, as shown above in Fig 3C. Responses in the naming blocks

Table 4. Debriefing responses by subject, paraphrased as they were reported to the experimenter. When subjects gave specific examples of color terms used as memory aids, they are reported here.

Subject	Debriefing: strategy during delay block
1	Used two-color words (e.g. 'blue-green') to help remember target.
2	Said the name of the color (e.g. 'periwinkle', 'yellow-green') during some delays.
3	Visualized color during delays.
4	Used verbal cues.
5	Used verbal cues ('blueish', 'green', etc.).
6	Used labels (e.g. 'forest green'), but reported feeling that it did not help much.
7	Tried to relate each target to something in nature (e.g. 'grass green').
8	Tried to use words (e.g. 'seafoam', 'forest green', 'mauve', etc.).
9	For first 10 trials, used the labels 'teal', 'grass green', and 'purple' as bases, and modified to the specific target (e.g. 'greener teal'). Subject reported finding this initial system too difficult, and just tried to picture the color in subsequent trials.
10	Associated each target with word (e.g. 'violet', 'olive green', 'forest green').
11	Mentally pictured target color.
12	Named color (e.g. 'teal', 'turquoise') and what "scheme" (e.g. 'orange') it was. Sometimes used a comparison to the previous trial ("a little more orange than previous target", etc.).
13	Named general color (e.g. 'blue', 'green'), and then pictured what shade it was.
14	Subject reported using no strategy other than mentally visualizing the color.
15	Subject reported visualizing the color and taking a mental snapshot of it.
16	Picked color that was closest to what was seen and graded degree to which it was similar (e.g. 'lighter', 'darker').
17	Mentally described target color (e.g. 'sky blue', 'olive green').
18	Used verbal descriptors for color (e.g. 'aquamarine', 'olive green').
19	Named color and gave mental description (e.g. 'teal').
20	No specific strategy reported.

doi:10.1371/journal.pone.0158725.t004

were self-paced. Naming blocks always followed reconstruction blocks, to ensure that repeated exposure to the color terms *green* and *blue* did not bias responses during reconstruction.

The experiment was presented in Matlab version 7.11.0 (R2010b) using Psychtoolbox (version 3) [39–41]. The experiment was conducted in a dark, sound-attenuated booth on an LCD monitor that supported 24-bit color. The monitor had been characterized using a Minolta CS100 colorimeter. A chin rest was used to ensure that each subject viewed the screen from a constant position; when in position, the base of the subject's chin was situated 30 cm from the screen.

As part of debriefing after testing was complete, each subject was asked to report any strategies they used during the delay block to help them remember the target color. Summaries of each response, as reported by the experimenter, are listed in Table 4.

Color spectrum. We wished to consider our stimuli along a 1-dimensional spectrum such that distance between two colors on that spectrum approximates the perceptual difference between those colors. To this end, we first converted our stimuli to CIELAB color space. CIELAB is a 3-dimensional color space designed "in an attempt to provide coordinates for colored stimuli so that the distance between the coordinates of any two stimuli is predictive of the perceived color difference between them" (p. 202 of [42]). The conversion to CIELAB was done according to the equations on pp. 167-168 of Wyszecki and Stiles (1982) [38], assuming 2 degree observer and D65 illuminant. For each pair of neighboring colors in the set of 677 colors of our color wheel, we measured the distance (ΔE) betwen these two colors in CIELAB space. We then arranged all colors along a 1-dimensional spectrum that was scaled to length 1, such

that the distance between each pair of neighboring colors along that spectrum was proportional to the CIELAB ΔE distance between them. This CIELAB-based 1-dimensional spectrum was used for our analyses in Study 1, and an analogous spectrum for a different set of colors was used for our analyses in Studies 2 and 3.

Statistical analysis. As a result of the experiment detailed above, we obtained bias data from 20 participants, for each of 19 hues (the medium range), for 5 trials per hue per participant, in each of the simultaneous and delayed conditions. For analysis purposes, we restricted attention to the focused range of stimuli (15 hues), in order to consider a region of the spectrum that is well-centered with respect to *green* and *blue*, as we are primarily interested in bias that may be induced by these two categories. We wished to determine whether the magnitude of the bias differed as a function of the simultaneous vs. delayed condition, whether the magnitude of the bias varied as a function of hue, and whether there was an interaction between these two factors. To answer those questions, we conducted a 2 (condition: simultaneous vs. delayed) × 15 (hues) repeated measures analysis of variance (ANOVA), in which the dependent measure was the absolute value of the reproduction bias (reproduced hue minus target hue), averaged across trials for a given participant at a given target hue in a given condition. The ANOVA included an error term to account for across-subject variability. We found a main effect of condition, with greater bias magnitude in the delayed than in the simultaneous condition [$F(1, 19) = 61.61$, $p < 0.0001$], a main effect of hue [$F(14, 266) = 4.565$, $p < 0.0001$], and an interaction of hue and condition [$F(14, 266) = 3.763$, $p < 0.0001$]. All hue calculations were relative to the CIELAB-based spectrum detailed in the preceding section.

We then conducted paired t-tests at each of the target hues, comparing each participant's bias magnitude for that hue (averaged over trials) in the simultaneous condition vs. the delayed condition. Blue asterisks at the top of Fig 4 mark hues for which the paired t-test returned $p < 0.05$ when applying Bonferroni corrections for multiple comparisons.

Modeling procedure. We considered four models in accounting for color reconstruction in English speakers: the null model, a 1-category model for which the category was *green*, a 1-category model for which the category was *blue*, and a 2-category model based on both *green* and *blue*.

We fit these models to the data in two steps. We first fit any category parameters (the means μ_c and variances σ_c^2 for any categories *c*) to the naming data. We then fit the one remaining free parameter (σ_m^2), which captures the uncertainty of fine-grained memory, to the bias data, without further adjusting the category parameters. We specify each of these two steps below.

We fit a Gaussian function to the goodness naming data for *green*, and another Gaussian function to the data for *blue*, using maximum likelihood estimation. The fitted Gaussian functions can be seen, together with the data to which they were fit, in the top panel of Fig 4. This process determined values for the category means μ_c and category variances σ_c^2 for the two categories *green* and *blue*.

For each of the four variants of the category adjustment model outlined above (null, 1-category green, 1-category blue, and 2-category), we retained the category parameter settings resulting from the above fit to the naming data. We then obtained a value for the one remaining free parameter σ_m^2, corresponding to the uncertainty of fine-grained memory, by fitting the model to the bias data via maximum likelihood estimation, without further adjusting the category parameters.

Study 2: Color discrimination across languages

Empirical data. The empirical data considered for this study were drawn from two sources: the study of 2AFC color discrimination by speakers of Berinmo and English in

Table 5. Hues for probed stimulus pairs within and across the English *blue-green*, Berinmo *wor-nol*, and Himba *dumbu-burou* boundaries. Any stimulus pair that includes a boundary color is considered to be a cross-category pair. All hues are at value (lightness) level 5, and chroma (saturation) level 8. 1s denotes a 1-step pair; 2s denotes a 2-step pair.

English *blue-green* stimuli (boundary color = 7.5BG)	
within	across
5B-2.5B (1s)	7.5BG-5BG (1s)
5B-10BG (2s)	2.5B-7.5BG (2s)
5BG-2.5BG (1s)	10BG-7.5BG (1s)
5BG-10G (2s)	10BG-5BG (2s)
Berinmo *wor-nol* stimuli (boundary color = 5GY)	
within	across
7.5Y-10Y (1s)	5GY-7.5GY (1s)
7.5Y-2.5GY (2s)	10Y-5GY (2s)
7.5GY-10GY (1s)	2.5GY-5GY (1s)
7.5GY-2.5G (2s)	2.5GY-7.5GY (2s)
Himba *dumbu-burou* stimuli (boundary color = 7.5GY)	
within	across
7.5Y-10Y (1s)	5GY-7.5GY (1s)
7.5Y-2.5GY (2s)	2.5GY-7.5GY (2s)
2.5GY-5GY (1s)	7.5GY-10GY (1s)
10Y-5GY (2s)	7.5GY-2.5G (2s)

doi:10.1371/journal.pone.0158725.t005

Experiment 6a of Roberson et al. (2000) [9], and the study of 2AFC color discrimination by speakers of Himba and English in Experiment 3b of Roberson et al. (2005) [10]. In both studies, two sets of color stimuli were considered, all at value (lightness) level 5, and chroma (saturation) level 8. Both sets varied in hue by increments of 2.5 Munsell hue steps. The first set of stimuli was centered at the English *green-blue* boundary (hue 7.5BG), and contained the following seven hues: 10G, 2.5BG, 5BG, 7.5BG, 10BG, 2.5B, 5B. The second set of stimuli was centered at the Berinmo *wor-nol* boundary (hue 5GY), and contained the following seven hues: 7.5Y, 10Y, 2.5GY, 5GY, 7.5GY, 10GY, 2.5G. Stimuli in the set that crossed an English category boundary all fell within a single category in Berinmo (*nol*) and in Himba (*burou*), and stimuli in the set that crossed a Berinmo category boundary also crossed a Himba category boundary (*dumbu-burou*) but all fell within a single category in English (*green*), according to naming data in Fig 1 of Roberson et al. (2005) [10]. Based on specifications in the original empirical studies [9, 10], we took the pairs of stimuli probed to be those presented in Table 5.

Based on naming data in Fig 1 of Roberson et al. 2005 [10], we took the prototypes of the relevant color terms to be:

English *green* prototype = 10GY

English *blue* prototype = 10B

Berinmo *wor* prototype = 5Y

Berinmo *nol* prototype = 5G

Himba *dumbu* prototype = 5Y

Himba *burou* prototype = 10G

Fig 6 above shows a spectrum of hues ranging from the Berinmo *wor* prototype (5Y) to the English *blue* prototype (10B) in increments of 2.5 Munsell hue steps, categorized according to each of the three languages we consider here. These Munsell hues were converted to xyY and then to CIELAB as above, and the positions of the hues on the spectrum were adjusted so that the distance between each two neighboring hues in the spectrum is proportional to the CIELAB ΔE distance between them. We use this CIELAB-based spectrum for our analyses below. The two shaded regions on each spectrum in Fig 6 denote the two target sets of stimuli identified above.

The discrimination data we modeled were drawn from Table 11 of Roberson et al. (2000:392) [9] and Table 6 of Roberson et al. (2005:400) [10].

Modeling procedure. We considered three variants of the 2-category model: an English *blue-green* model, a Berinmo *wor-nol* model, and a Himba *dumbu-burou* model. As in Study 1, we fit each model to the data in two steps. For each language's model, we first fit the category component of that model to naming data from that language. Because color naming differs across these languages, this resulted in three models with different category components. For each model, we then retained and fixed the resulting category parameter settings, and fit the single remaining parameter, corresponding to memory uncertainty, to discrimination data. We detail these two steps below.

For the naming data, we modeled the probability of applying category name c to stimulus i as:

$$p(c|i) \propto p(i|c)p(c) \propto f(i|c)p(c) \tag{13}$$

where $p(c)$ is assumed to be uniform, and $f(i|c)$ is a non-normalized Gaussian function corresponding to category c, with mean μ_c and variance σ_c^2. There were two categories c for each model, e.g. *wor* and *nol* in the case of the Berinmo model. Category means μ_c were set to the corresponding category prototypes shown above (e.g. μ_c for Berinmo *nol* corresponded to 5G), and category variances σ_c^2 were left as free parameters. We then adjusted these free category variances to reproduce the empirical boundary between the two categories c_1 and c_2 for that language, as follows. Sweeping from left (c_1) to right (c_2), we took the model's boundary between c_1 and c_2 to be the first position i on the spectrum for which $p(c_1|i) \leq p(c_2|i)$; we refer to this as the *model crossover point*. We measured the distance in the CIELAB-based spectrum between the model crossover point and the empirical category boundary, and adjusted the category variances $\sigma_{c_1}^2$ and $\sigma_{c_2}^2$ so as to minimize that distance. This was done separately for each language's model. Fig 6 shows the resulting fits of category components to naming data for each of the three languages.

We then simulated performance in the 2AFC discrimination task for each stimulus pair in Table 5, by each model, as follows. Given a pair of stimuli, one stimulus was taken to be the target t and therefore held in memory, and the other taken to be the distractor d. We took the reconstruction r for the target stimulus t to be the expected value of the posterior for the 2-category model:

$$r = E[t|M] \tag{14}$$

We then measured, along the hue spectrum in question, the distance $dist(r, t)$ between the reconstruction r and the target t, and the distance $dist(r, d)$ between the reconstruction r and the distractor d. We converted each of these two distances to a similarity score:

$$sim(i,j) = \exp(-dist(i,j)) \tag{15}$$

Table 6. Hues for probed stimulus pairs within and across the English *green-blue* boundary for Study 3, from Table 1 of Roberson and Davidoff (2000). Any stimulus pair that includes a boundary color is considered to be a cross-category pair. All hues are at value (lightness) level 4, and chroma (saturation) level 8. 1s denotes a 1-step pair; 2s denotes a 2-step pair.

English *green-blue* stimuli (boundary color = 7.5BG)	
**within**	**across**
5B-2.5B (1s)	10BG-7.5BG (1s)
5B-10BG (2s)	2.5B-7.5BG (2s)
2.5BG-10G (1s)	7.5BG-5BG (1s)
5BG-10G (2s)	10BG-5BG (2s)

doi:10.1371/journal.pone.0158725.t006

and modeled the proportion correct choice as:

$$p = \frac{sim(r,t)}{\sum_{i\in\{t,d\}} sim(r,i)} \qquad (16)$$

These equations are based on Luce's [43] (pp. 113-114) model of choice behavior. For each pair of stimuli, each stimulus was once taken to be the target, and once taken to be the distractor, and the results averaged to yield a mean discrimination score for that pair. Scores were then averaged across all pairs listed as within-category pairs, and separately for all pairs listed as cross-category pairs. These scores were range-matched to the empirical data, in an attempt to correct for other factors that could affect performance, such as familiarity with such tasks, etc.; such external factors could in principle differ substantially across participant pools for the three languages modeled. We measured MSE between the model output and the data so treated, and adjusted the remaining parameter σ_m^2, corresponding to memory uncertainty, so as to minimize this MSE. This entire process was conducted two times. The first time, each language's model was fit to that same language's discrimination data. Then, to test whether native-language categories allow a better fit than the categories of another language, we fit the Berinmo model to the English discrimination data (and vice versa), and the Himba model to the English discrimination data (and vice versa).

Study 3: Within-category effects

Empirical data. The empirical data considered for this study are those of Figs 2 (English *green/blue*, 10 second delay), 3 (Berinmo *wor/nol*), and 4 (Himba *dumbu/borou*) of Hanley and Roberson (2011) [36]. These data were originally published by Roberson and Davidoff (2000) [8], Roberson et al. (2000) [9], and Roberson et al. (2005) [10], respectively. The Berinmo and Himba stimuli and data were the same as in our Study 2, but the English stimuli and data reanalyzed by Hanley and Roberson (2011) [36] Fig 2 were instead drawn from Table 1 of Roberson and Davidoff (2000) [8], reproduced here in Table 6, and used for the English condition of this study. These stimuli for English were at lightness (value) level 4, rather than 5 as for the other two languages. We chose to ignore this difference for modeling purposes.

Modeling procedure. All modeling procedures were identical to those of Study 2, with the exception that GE (target = good exemplar) and PE (target = poor exemplar) cases were disaggregated, and analyzed separately.

Acknowledgments

We thank Roland Baddeley, Paul Kay, Charles Kemp, Steven Piantadosi, and an anonymous reviewer for their comments.

Author Contributions

Conceived and designed the experiments: EC YX JLA TLG TR. Performed the experiments: EC YX JLA. Analyzed the data: EC YX JLA. Wrote the paper: TR EC YX.

References

1. Sapir E. The status of linguistics as a science. Language. 1929; 5:207–214. doi: 10.2307/409588
2. Whorf BL. Science and linguistics. In: Carroll JB, editor. Language, Thought, and Reality: Selected Writings of Benjamin Lee Whorf. MIT Press; 1956. p. 207–219.
3. Berlin B, Kay P. Basic color terms: Their universality and evolution. University of California Press; 1969.
4. Kay P, Regier T. Resolving the question of color naming universals. Proceedings of the National Academy of Sciences. 2003; 100:9085–9089. doi: 10.1073/pnas.1532837100
5. Kay P, Berlin B, Maffi L, Merrifield WR, Cook R. The World Color Survey. CSLI Publications; 2009.
6. Brown RW, Lenneberg EH. A study in language and cognition. The Journal of Abnormal and Social Psychology. 1954; 49:454–462. doi: 10.1037/h0057814
7. Kay P, Kempton W. What is the Sapir-Whorf hypothesis? American Anthropologist. 1984; 86:65–79. doi: 10.1525/aa.1984.86.1.02a00050
8. Roberson D, Davidoff J. The categorical perception of colors and facial expressions: The effect of verbal interference. Memory and Cognition. 2000; 28:977–986. doi: 10.3758/BF03209345 PMID: 11105523
9. Roberson D, Davies I, Davidoff J. Color categories are not universal: Replications and new evidence from a stone-age culture. Journal of Experimental Psychology: General. 2000; 129:369–398. doi: 10.1037/0096-3445.129.3.369
10. Roberson D, Davidoff J, Davies IRL, Shapiro LR. Color categories: Evidence for the cultural relativity hypothesis. Cognitive Psychology. 2005; 50:378–411. doi: 10.1016/j.cogpsych.2004.10.001 PMID: 15893525
11. Gilbert A, Regier T, Kay P, Ivry R. Whorf hypothesis is supported in the right visual field but not the left. Proceedings of the National Academy of Sciences. 2006; 103:489–494. doi: 10.1073/pnas.0509868103
12. Winawer J, Witthoft N, Frank MC, Wu L, Wade AR, Boroditsky L. Russian blues reveal effects of language on color discrimination. Proceedings of the National Academy of Sciences. 2007; 104:7780–7785. doi: 10.1073/pnas.0701644104
13. Roberson D, Pak H, Hanley JR. Categorical perception of colour in the left and right visual field is verbally mediated: Evidence from Korean. Cognition. 2008; 107:752–762. doi: 10.1016/j.cognition.2007.09.001 PMID: 17931614
14. Drivonikou GV, Kay P, Regier T, Ivry RB, Gilbert AL, Franklin A, et al. Further evidence that Whorfian effects are stronger in the right visual field than the left. Proceedings of the National Academy of Sciences. 2007; 104:1097–1102. doi: 10.1073/pnas.0610132104
15. Brown AM, Lindsey DT, Guckes KM. Color names, color categories, and color-cued visual search: Sometimes, color perception is not categorical. Journal of Vision. 2011; 11:2. doi: 10.1167/11.12.2 PMID: 21980188
16. Witzel C, Gegenfurtner KR. Is there a lateralized category effect for color? Journal of Vision. 2011; 11 (12). doi: 10.1167/11.12.16 PMID: 22019716
17. Wright O, Davies IRL, Franklin A. Whorfian effects on colour memory are not reliable. The Quarterly Journal of Experimental Psychology. 2015; 68:745–758. doi: 10.1080/17470218.2014.966123 PMID: 25230782
18. Huttenlocher J, Hedges LV, Duncan S. Categories and particulars: Prototype effects in estimating spatial location. Psychological Review. 1991; 98:352–376. doi: 10.1037/0033-295X.98.3.352 PMID: 1891523
19. Huttenlocher J, Hedges LV, Vevea JL. Why do categories affect stimulus judgment? Journal of Experimental Psychology: General. 2000; 129:220–241. doi: 10.1037/0096-3445.129.2.220

20. Bae GY, Olkkonen M, Allred SR, Flombaum JI. Why some colors appear more memorable than others: A model combining categories and particulars in color working memory. Journal of Experimental Psychology: General. 2015; 144:744–763. doi: 10.1037/xge0000076
21. Persaud K, Hemmer P. The influence of knowledge and expectations for color on episodic memory. In: Bello P, Guarini M, McShane M, Scassellati B, editors. Proceedings of the 36th Annual Meeting of the Cognitive Science Society. Cognitive Science Society; 2014. p. 1162–1167.
22. Yuille AL, Bülthoff HH. Bayesian decision theory and psychophysics. In: Knill DC, Richards W, editors. Perception as Bayesian Inference. Cambridge University Press; 1996. p. 123–162.
23. Jacobs RA. What determines visual cue reliability? Trends in Cognitive Sciences. 2002; 6:345–350. doi: 10.1016/S1364-6613(02)01948-4 PMID: 12140085
24. Ernst MO, Banks MS. Humans integrate visual and haptic information in a statistically optimal fashion. Nature. 2002; 415:429–433. doi: 10.1038/415429a PMID: 11807554
25. Knill DC, Pouget A. The Bayesian brain: The role of uncertainty in neural coding and computation. Trends in Neurosciences. 2004; 27:712–719. doi: 10.1016/j.tins.2004.10.007 PMID: 15541511
26. Holden MP, Newcombe NS, Shipley TF. Location memory in the real world: Category adjustment effects in 3-dimensional space. Cognition. 2013; 128:45–55. doi: 10.1016/j.cognition.2013.02.016 PMID: 23578687
27. Hemmer P, Steyvers M. A Bayesian account of reconstructive memory. Topics in Cognitive Science. 2009; 1:189–202. doi: 10.1111/j.1756-8765.2008.01010.x PMID: 25164805
28. Feldman NH, Griffiths TL, Morgan JL. The influence of categories on perception: Explaining the perceptual magnet effect as optimal statistical inference. Psychological Review. 2009; 116:752–782. doi: 10.1037/a0017196 PMID: 19839683
29. Lupyan G. From Chair To 'Chair:' A representational shift account of object labeling effects on memory. Journal of Experimental Psychology: General. 2008; 137:348–369. doi: 10.1037/0096-3445.137.2.348
30. Lupyan G. Linguistically modulated perception and cognition: The label-feedback hypothesis. Frontiers in Psychology. 2012; 3:54. doi: 10.3389/fpsyg.2012.00054 PMID: 22408629
31. Hu Z, Hanley JR, Zhang R, Liu Q, Roberson D. A conflict-based model of color categorical perception: Evidence from a priming study. Psychonomic Bulletin and Review. 2014; 21:1214–1223. doi: 10.3758/s13423-014-0603-8 PMID: 24638827
32. Roberson D, Damjanovic L, Pilling M. Categorical perception of facial expressions: Evidence for a "category adjustment" model. Memory and Cognition. 2007; 35:1814–1829. doi: 10.3758/BF03193512 PMID: 18062556
33. Goldstone RL. Effects of categorization on color perception. Psychological Science. 1995; 6:298–304. doi: 10.1111/j.1467-9280.1995.tb00514.x
34. Hemmer P, Persaud K. Interaction between categorical knowledge and episodic memory across domains. Frontiers in Psychology. 2014; 5:584. doi: 10.3389/fpsyg.2014.00584 PMID: 24966848
35. Hemmer P, Persaud K, Kidd C, Piantadosi S. Inferring the Tsimane's use of color categories from recognition memory. In: Noelle DC, Dale R, Warlaumont AS, Yoshimi J, Matlock T, Jennings CD, et al., editors. Proceedings of the 37th Annual Meeting of the Cognitive Science Society. Cognitive Science Society; 2015. p. 896–901.
36. Hanley JR, Roberson D. Categorical perception effects reflect differences in typicality on within-category trials. Psychonomic Bulletin and Review. 2011; 18:355–363. doi: 10.3758/s13423-010-0043-z PMID: 21327385
37. Regier T, Kay P, Khetarpal N. Color naming reflects optimal partitions of color space. Proceedings of the National Academy of Sciences. 2007; 104:1436–1441. doi: 10.1073/pnas.0610341104
38. Wyszecki G, Stiles WS. Color science: Concepts and methods, quantitative data and formulae. Wiley; 1982.
39. Brainard D. The Psychophysics Toolbox. Spatial Vision. 1997; 10:433–436. doi: 10.1163/156856897X00357 PMID: 9176952
40. Pelli D. The VideoToolbox software for visual psychophysics: Transforming numbers into movies. Spatial Vision. 1997; 10:437–442. doi: 10.1163/156856897X00366 PMID: 9176953
41. Kleiner M, Brainard D, Pelli D. What's new in Psychtoolbox-3? Perception. 2007; 36:ECVP Abstract Supplement.
42. Brainard DH. Color appearance and color difference specification. In: Shevell SK, editor. The science of color: Second edition. Elsevier; 2003. p. 191–216.
43. Luce RD. Detection and recognition. In: Luce RD, Bush RR, Galanter E, editors. Handbook of mathematical psychology. Wiley; 1963. p. 103–189.
